# Supplementary material for: Temperature Dependence of Platelet Metabolism
Source: Metabolites. 2024 Jan 26;14(2):91. doi: 10.3390/metabo14020091 (PMC10890334; doi:10.3390/metabo14020091)
Supplement: Supplementary file 1 [file metabolites-14-00091-s001.zip › Supplementary_Material_S1.pdf]

Supporting information for “Temperature dependence of platelet metabolism”

Freyr Jóhannsson, James T. Yurkovich, Steinn Guðmundsson, Ólafur E. Sigurjónsson, and Óttar Rolfsson

Contents

Table S1. Platelet concentrate quality control analysis measured with a blood gas analyzer. 2

Table S2. Platelet concentrate quality control analysis measured with a hematology analyzer. 3

Figure S1. Platelet concentrate pH measured with a blood gas analyzer 4

Figure S2. Metabolic flux within the TCA cycle decreases independently of the glucose uptake rate. 5

Table S1. Platelet concentrate quality control analysis measured with a blood gas analyzer.

Temperature	Time (hours)	pH	pCO2 (mmHg)	pO2 (mmHg)	K+ (mM)	Na+ (mM)	Cl- (mM)
4°C	24	6.91	25.9	143	4.8	158	76
4°C	48	6.92	19.7	143	5.0	157	76
4°C	108	6.86	12.4	163	5.2	157	76
4°C	216	6.74	8.1	241	5.3	158	76
4°C	324	6.69	6.2	259	5.4	158	77
4°C	432	6.71	0	258	5.6	159	77
4°C	552	6.69	0	256	5.7	160	77
13°C	24	6.98	19.2	147	4.3	159	76
13°C	48	6.97	14.7	145	4.3	159	76
13°C	60	6.94	13.6	141	4.4	158	76
13°C	120	6.85	10.3	149	4.8	158	76
13°C	180	6.97	8.0	172	5.0	158	76
13°C	240	7.05	6.9	177	5.2	159	76
13°C	312	7.15	NA	192	5.4	159	76
22°C	24	7.05	16.2	137	4.2	159	75
22°C	48	7.08	13.6	137	4.3	159	76
22°C	72	7.06	14.4	140	4.4	159	76
22°C	96	7.00	12.1	143	4.5	160	77
22°C	120	7.07	9.9	144	4.7	161	76
22°C	144	7.17	8.5	139	4.9	161	77
22°C	168	7.26	7.4	147	5.0	161	77
37°C	3	6.95	26.5	60.5	4.1	159	76
37°C	6	7.00	21.7	81.9	4.2	159	76
37°C	24	7.08	15.1	67	4.6	161	76
37°C	27	7.08	14.8	112	4.6	161	77
37°C	30	7.11	13.2	106	4.6	162	77
37°C	48	7.29	9.0	114	5.0	164	78
37°C	51	7.33	8.6	98.4	5.1	164	78

Table S2. Platelet concentrate quality control analysis measured with a hematology analyzer.

Temperature	Time (hours)	PLT (10e9/L)	WBC (10e9/L)	RBC (10e12/L)	MPV (fL)
4°C	24	1589	0.01	0.14	11.2
4°C	48	1638	0.00	0.14	11.2
4°C	108	1643	0.02	0.13	11.1
4°C	216	1627	0.03	0.14	11.1
4°C	324	1457	0.06	0.12	11.0
4°C	432	1457	0.08	0.11	10.8
4°C	552	1366	0.40	0.09	10.7
13°C	24	1549	0.02	0.14	11.1
13°C	48	1608	0.01	0.16	11.3
13°C	60	1662	0.01	0.18	11.4
13°C	120	1599	0.02	0.20	11.7
13°C	180	1591	0.01	0.20	11.9
13°C	240	1544	0.00	0.22	12.1
13°C	312	1433	0.01	0.30	12.6
22°C	24	1340	0.01	0.00	10.2
22°C	48	1433	0.01	0.11	10.3
22°C	72	1502	0.01	0.13	10.5
22°C	96	1624	0.02	0.16	10.7
22°C	120	1620	0.02	0.18	11.0
22°C	144	1629	0.01	0.19	11.1
22°C	168	1554	0.01	0.22	11.5
37°C	3	1451	0.02	0.08	10.0
37°C	6	1574	0.02	0.09	9.9
37°C	24	1476	0.02	0.14	10.0
37°C	27	1750	0.05	0.25	10.7
37°C	30	1316	0.02	0.11	10.0
37°C	48	1198	0.02	0.11	10.1
37°C	51	1208	0.06	0.12	10.1

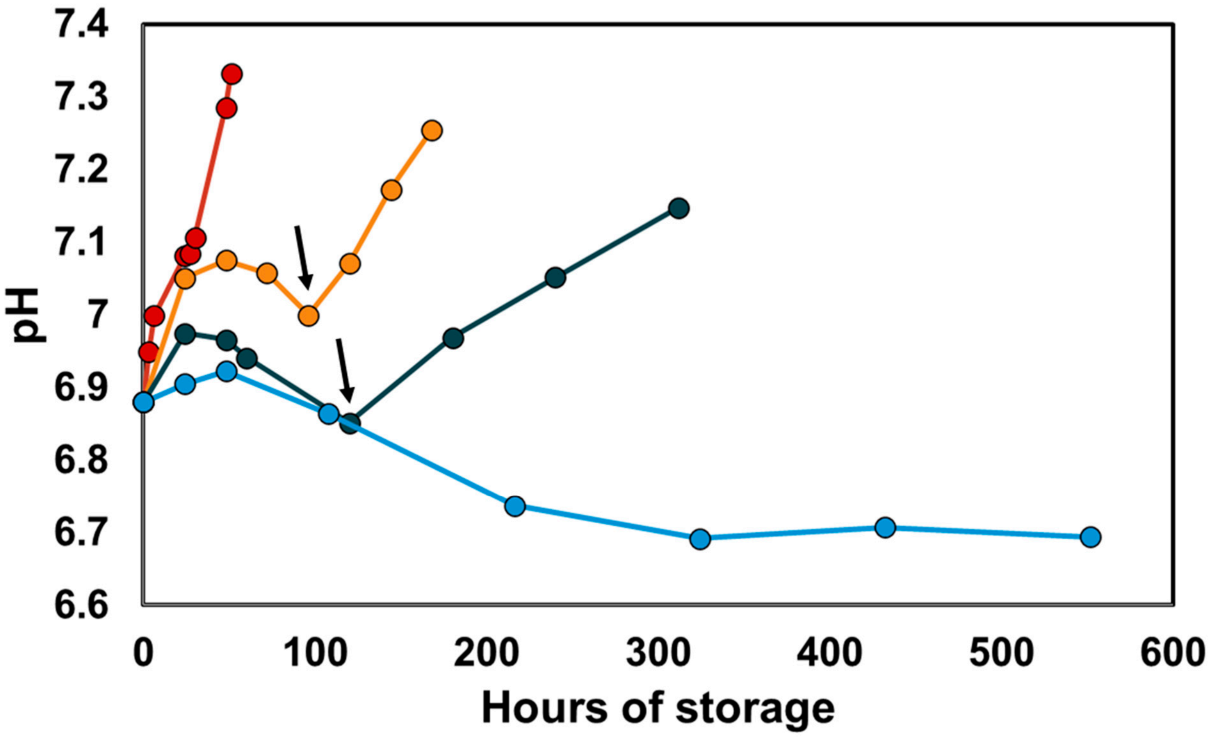

Figure S1. Platelet concentrate pH measured with a blood gas analyzer. The platelets were stored at 4°C (blue), 13°C (grey), 22°C (yellow), and 37°C (red). The arrows indicate the time point at which the glucose was depleted in the platelet concentrates stored at 22°C and 13°C.

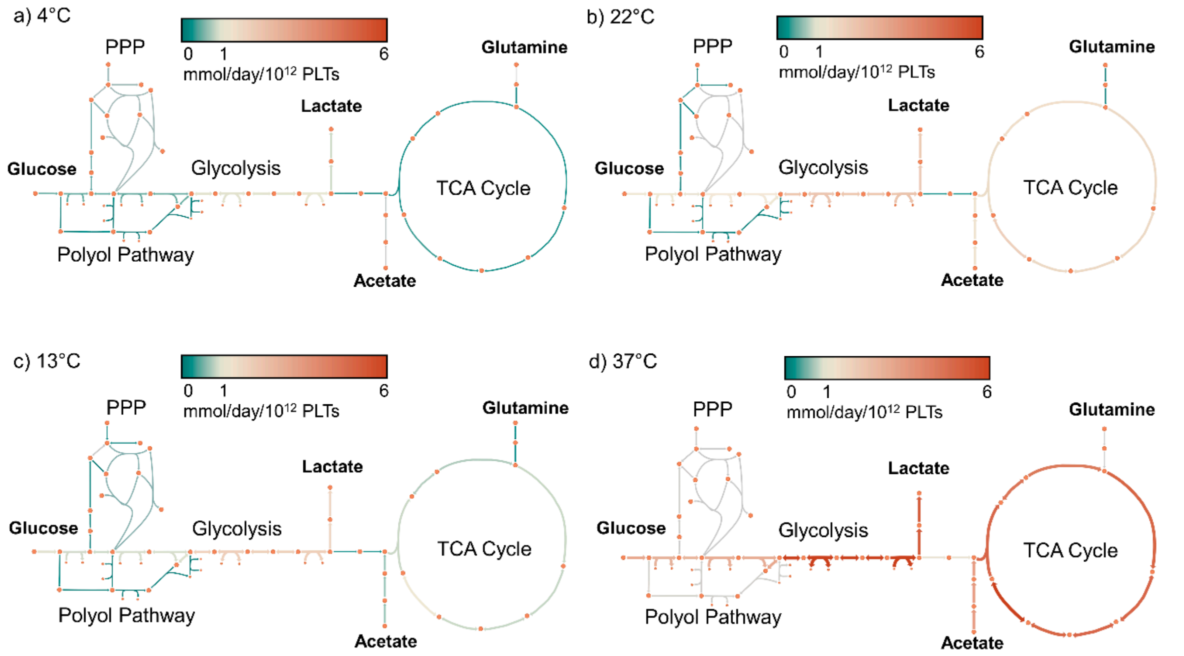

Figure S2. Metabolic flux within the TCA cycle decreases independently of the glucose uptake rate. Flux maps (a-d) showing the calculated flux in mmol(gdw)$^{-1}$(h)$^{-1}$.
